# Placental Vessel Segmentation Using Pix2pix Compared to U-Net

**DOI:** 10.3390/jimaging9100226

**Published:** 2023-10-16

**Authors:** Anouk van der Schot, Esther Sikkel, Marèll Niekolaas, Marc Spaanderman, Guido de Jong

**Affiliations:** 1 Obstetrics & Gynecology, Radboud University Medical Center, 6525 GA Nijmegen, The Netherlands; 2 Obstetrics & Gynecology, Maastricht University Medical Center, 6229 ER Maastricht, The Netherlands; 3 Department of GROW, School for Oncology and Reproduction, Maastricht University, 6229 ER Maastricht, The Netherlands; 4 3D Lab, Radboud University Medical Center, 6525 GA Nijmegen, The Netherlands

**Keywords:** fetal surgery, fetoscopy, twin-to-twin transfusion syndrome, vessel segmentation, generative artificial intelligence

## Abstract

Computer-assisted technologies have made significant progress in fetoscopic laser surgery, including placental vessel segmentation. However, the intra- and inter-procedure variabilities in the state-of-the-art segmentation methods remain a significant hurdle. To address this, we investigated the use of conditional generative adversarial networks (cGANs) for fetoscopic image segmentation and compared their performance with the benchmark U-Net technique for placental vessel segmentation. Two deep-learning models, U-Net and pix2pix (a popular cGAN model), were trained and evaluated using a publicly available dataset and an internal validation set. The overall results showed that the pix2pix model outperformed the U-Net model, with a Dice score of 0.80 [0.70; 0.86] versus 0.75 [0.0.60; 0.84] (*p*-value < 0.01) and an Intersection over Union (IoU) score of 0.70 [0.61; 0.77] compared to 0.66 [0.53; 0.75] (*p*-value < 0.01), respectively. The internal validation dataset further validated the superiority of the pix2pix model, achieving Dice and IoU scores of 0.68 [0.53; 0.79] and 0.59 [0.49; 0.69] (*p*-value < 0.01), respectively, while the U-Net model obtained scores of 0.53 [0.49; 0.64] and 0.49 [0.17; 0.56], respectively. This study successfully compared U-Net and pix2pix models for placental vessel segmentation in fetoscopic images, demonstrating improved results with the cGAN-based approach. However, the challenge of achieving generalizability still needs to be addressed.

## 1. Introduction

Fetoscopic laser surgery is the current treatment for twin-to-twin transfusion syndrome (TTTS) [1]. In this rare pregnancy condition, placental anastomoses cause an imbalance in the shared blood circulation between monochorionic twin fetuses. The fetoscopic laser procedure is challenging, mainly due to the fetoscope’s limited field of view, leading to increased procedural time and incomplete ablation of anastomoses. Computer-assisted technologies, like placental vessel segmentation and fetoscopic image stitching algorithms for the fetal environment, have been developed to overcome the limited field of view challenges by supporting the identification of the placental vessels and anastomoses [2].

Placental vessel segmentation has shown promising opportunities for vessel-based fetoscopic image stitching regarding robustness in qualitative and quantitative comparison [3]. This method stitches successive fetoscopic images to artificially expand the field of view guided by segmented placental vessel maps. Moreover, accurate placental vessel segmentation may have significant advantages for improved visualization of the fetoscopic environment and assisting fetal surgeons in the localization of anastomoses.

Nevertheless, the investigation into techniques for segmenting fetal images remains relatively limited. While efforts have been made to histopathological images [4,5,6,7,8], the application of these methods to fetoscopic images has only been marginally explored. Here, placental vessel segmentation is of most interest and is currently a commonly reoccurring topic in the literature. Most proposed methods use a U-Net-based architecture, a model developed for biomedical image segmentation to perform patch-based segmentation [3,8,9,10]. Through the fetoscopic placental vessel segmentation and registration (FetReg2021) challenge, a benchmark method for fetoscopic placental vessel segmentation and registration has been provided for future research [4,11]. Nevertheless, the intra- and inter-procedure variability remains a significant hurdle [4].

In recent years, segmentation methods based on conditional generative adversarial networks (cGANs), including pix2pix, have been increasingly explored in medical image research [12]. Hence, GANs have proven their value in retinal vessel segmentation by combining GANs with classical architectures like U-Net to improve performance [13,14]. Inspired by these applications, we experimentally evaluated the performance of a GAN framework when applied to fetoscopic segmentation tasks and compared these results with the FetReg2021 benchmark method, which outperformed other solutions. We explored the use of a cGAN model, i.e., pix2pix, for fetoscopic image segmentation and compared this with the benchmark technique, i.e., U-Net, for placental vessel segmentation.

## 2. Materials and Methods

### 2.1. Image Acquisition

The publicly available fetoscopy placental dataset of Bano et al., containing 483 frames with ground-truth vessel segmentation annotations taken from six different in vivo fetoscopic procedures, was used in this study [3]. In this dataset, the non-occluded frames, i.e., the frames where no fetus or tool is present, were selected for the ground truth vessel annotations. The performance of the vessel segmentation algorithm was evaluated with a second internal dataset. In a process approved by an institutional review board (case number 2018-4250), intraoperative videos were obtained from nine fetoscopic laser surgeries to treat TTTS. All videos were recorded using Straight Forward Telescopes (Karl Storz, Tuttlingen, Germany) with incorporated fiber optic light transmission. More specifically, a 2.0 mm HOPKINS^®^ II Straight Forward Telescope 0° (26008AA) and a 2.9 mm HOPKINS^®^ II Straight Forward Telescope 30° (26120BA) were used for posterior and anterior placentas, respectively. The fetoscopic image frames were saved with a resolution of 1920 × 1080 pixels with RGB color channels. A total of 476 fetoscopic frames were collected. The video frames were cropped to make the circular field of view from the fetoscope fully fit the frame and stored twice: a full-resolution version and a compromised version, meaning that the frame was downsized to 256 × 256 × 3 pixels. Ground truth annotations were manually annotated, creating a binary mask of the vasculature in each frame and further verified by a clinical expert to confirm correctness.

### 2.2. Network Architecture

#### 2.2.1. U-Net

U-Net is a fully convolutional network architecture for fast semantic segmentation and is the state-of-the-art segmentation model for biomedical images [15]. The main idea of the network is to add additional layers to a typical network and replace pooling operations with upsampling operators. The architecture consists of an encoder network followed by a decoder network, giving the U-shape. The encoder, the first half of the architecture, usually consists of a classification network to encode the input image into feature representations at multiple levels. The second half of the architecture, the decoder, consists of upsampling and concatenation followed by regular convolution operations to project the features onto the pixel space [15]. Like Bano et al., we used the sum of the binary cross-entropy loss and Jaccard loss (Appendix A.1.1) during training [3,4].

#### 2.2.2. Pix2pix cGAN

The pix2pix network contains two submodels, a U-Net type generator and a PatchGAN discriminator, which compete. In contrast to the general U-Net, the pix2pix generator has two methods to update its weights in the convolutional filters during training. First, like U-Net, the network uses an improved backpropagation through skip connections. Second, the external path compares the ground truth and fake images from the discriminator. As a result, the generator can create segmentation maps that closely resemble the target images [16]. The loss function of the pix2pix model is described as a combination of the L1 loss function and conditional adversarial loss (Appendix A.1.2).

#### 2.2.3. Training

Both models were built and trained in Python 3 on an Nvidia-SMI Quadro RTX 6000 GPU. The hyperparameters of the U-Net were set to the same value as described by Bano et al. [3]. The backbone was set at ResNet101, and the model was trained with a batch size of 1 for 1000 epochs, with early stopping. The learning rate was set to 3 × 10^−4^ with Adam optimizer and the combined loss as described in Section 2.2.1. For each iteration, the images were cropped with a size of 224 × 224, and random augmentation was applied (rotation, horizontal and vertical flip, and illumination intensity).

The pix2pix model was based on the PyTorch implementation of Isola et al. [16]. The generator architecture was set to a U-Net (256 × 256 input) architecture, and the basic model 70 × 70 PatchGAN was used for the discriminator. The model was trained with a batch size of 1 for 1000 epochs, where the learning rate was set to 1 × 10^−3^ and constant for the first 500 epochs. For the last 500 epochs, the learning rate linearly decayed to zero. Adam was used for optimization with fixed β values of 0.9 and 0.99. Data augmentation was applied by scaling and cropping at load time to 256 × 256 pixels.

Both models were validated on the left-out set of the six-folds. After that, both models were trained with the whole dataset (all six folds) and validated on our internal dataset.

#### 2.2.4. Evaluation Metrics

To compare the results with the literature, 6-fold cross-validation was used with the same folds as described in the original paper of Bano et al. [3]. The Dice score and mean Intersection over Union (IoU) (Jaccard Index) were used to evaluate the segmentation performance. The Dice score takes two times the area of overlap, divided by the total number of pixels in both images (Equation (1)) [17]. The IoU equals the area of overlap divided by the area of union (Equation (2)) [18].

Dice-score = (2 × |X∩Y|)/(|X| + |Y|)(1)

IoU = (X∩Y)/(X∪Y)(2)

For fair measurement, only pixels inside the circular field of view of the fetoscope were considered when computing the measures.

To determine whether or not there were significant differences between the tested models, the Wilcoxon signed rank test on the paired Dice and IoU scores with a significance level (*p*) equal to 0.05 was applied.

## 3. Results

We trained the U-Net model described by Bano et al. to investigate its reproducibility [3]. In addition, we trained the pix2pix model and evaluated these results on the test set for every fold. Finally, both models, i.e., the reproduced U-Net and pix2pix model, were validated with the internal dataset.

**Figure 1:**
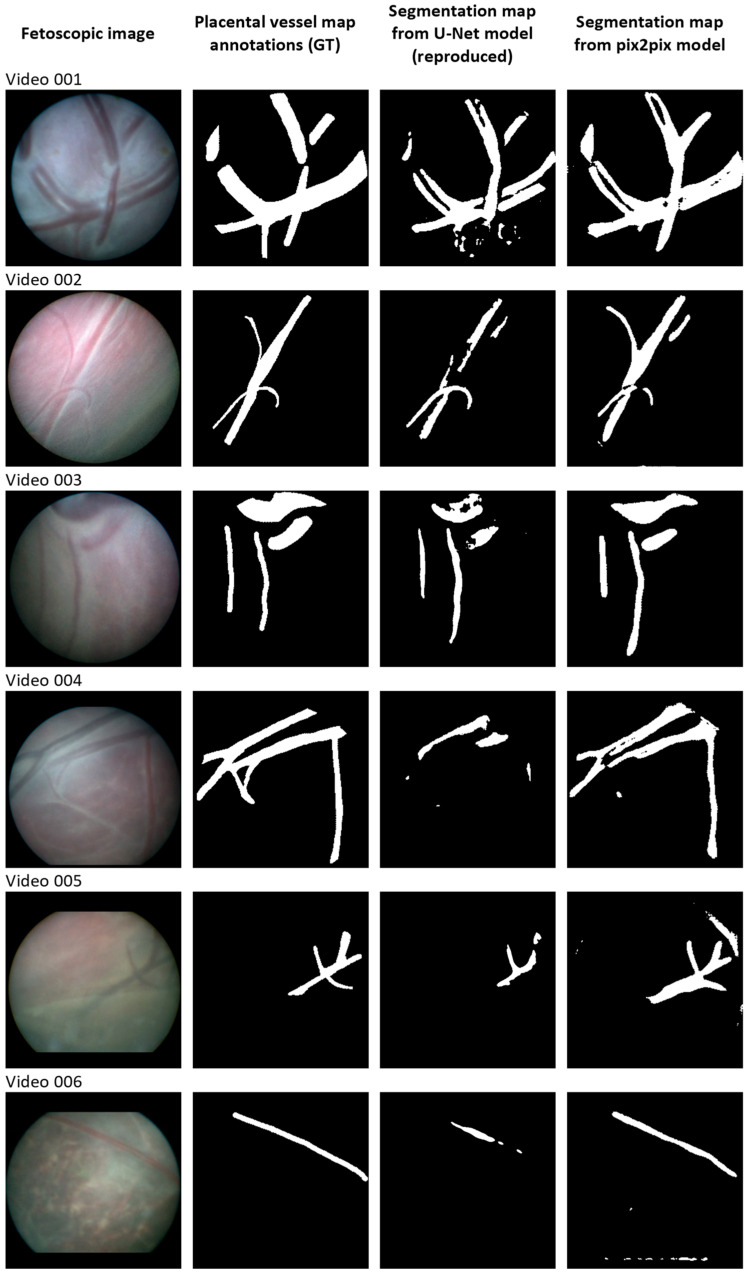
Qualitative results of U-Net and pix2pix model for placental vessel segmentation, compared with ground truth (GT).

**Figure 2:**
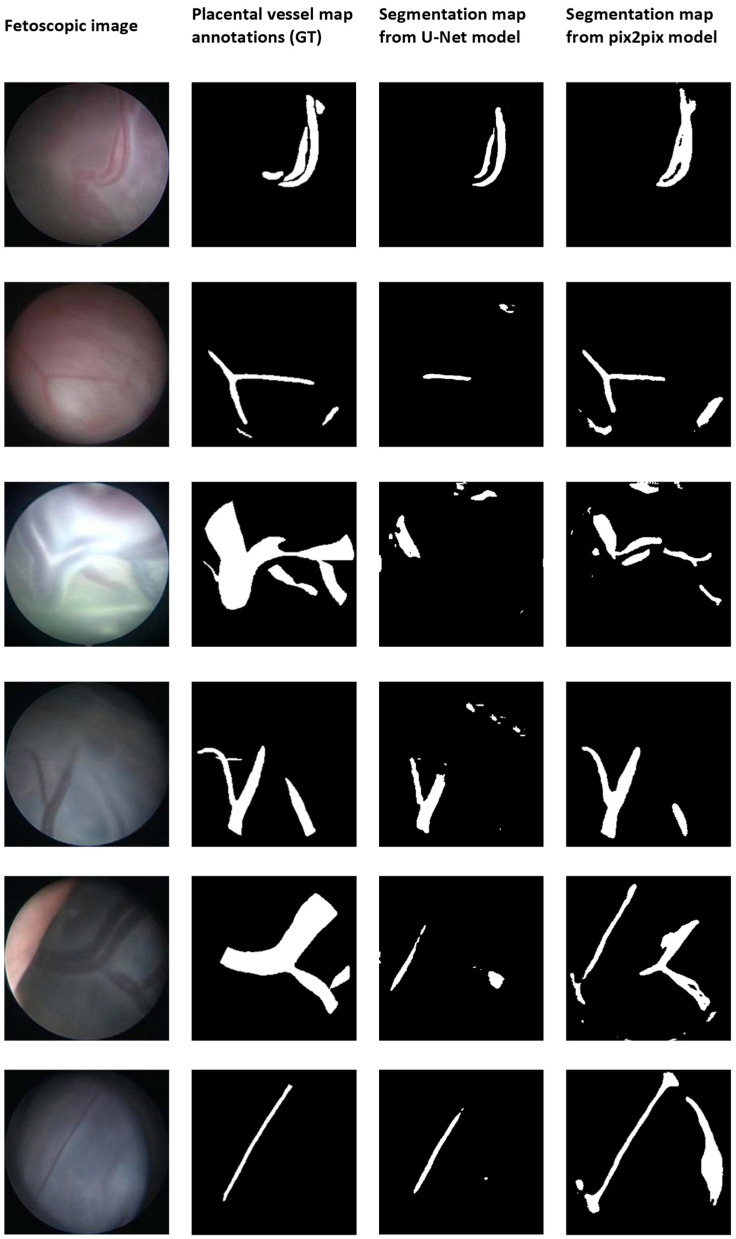
Validation of placental vessel segmentation models, i.e., the state-of-the-art U-Net and the proposed pix2pix model, on the intern dataset.

Figure 1 shows random examples of the segmentation results of both models. In addition, Figure 2 shows random samples of the validation results on our internal dataset. Each row shows the original image, the ground truth, and the segmentation results extracted from the test videos. Corresponding quantitative results, i.e., the median Dice score, IoU score, and interquartile intervals, are provided in Table 1. These scores were compared with the baseline findings of the original paper [3]. Note that we used the median due to the data not being normally distributed, while the original results were reported in mean and standard deviation.

The overall Dice scores resulted in a score of 0.80 [0.70; 0.86] for the pix2pix model, as opposed to the U-Net model’s overall score of 0.75 [0.0.60; 0.84] (*p*-value < 0.01). Regarding the overall Intersection over Union (IoU) score, the U-Net model achieved a value of 0.66 [0.53; 0.75], while the pix2pix model achieved a significantly higher score of 0.70 [0.61; 0.77] (*p*-value < 0.01). When considering the validation with the internal database, the Dice and IoU scores for the U-Net model were 0.53 [0.49; 0.64] and 0.49 [0.17; 0.56], respectively, whereas the pix2pix model obtained scores of 0.68 [0.53; 0.79] and 0.59 [0.49; 0.69] (*p*-value < 0.01), respectively. For both scores, the difference was significant (*p*-value < 0.01), where pix2pix outperformed the U-Net.

## 4. Discussion

Accurate placental vessel segmentation plays an essential role in a better understanding and visualization of the placental surface during fetal laser surgery. It has shown its potential in developing computer-assisted surgery, including vessel-based image registration, to artificially expand the field of view of the fetoscope [3]. Despite the introduction of public databases and a benchmark technique for research [3,4,11], the segmentation results still need improvement due to the high intra- and inter-variability in surgical procedures. In this study, we hypothesized that a cGAN architecture would provide better segmentation results for relatively small datasets than the benchmark technique, i.e., a general U-Net architecture.

In assessing the performance of a cGAN for placental vessel segmentation compared to a standard U-Net model, we initially examined the reproducibility of the benchmark U-Net model. Despite minor variability among different folds, we were able to reproduce the benchmark results as published in earlier studies [3,4,10,11], including a recent challenge organized as part of the MICCAI2021 Endoscopic Vision challenge [4]. Differences in models and backbones of the U-Net have been investigated by Casella et al. [10], who showed similar performance between the architectures and found a comparable mean IoU of 0.63 ± 0.19. Additionally, we evaluated both models using a novel internal dataset. On the one hand, we observed relatively low scores in Dice and IoU metrics, indicating the models’ lack of generalizability. Significant intra-procedure variability persisted. On the other hand, the proposed pix2pix model has significantly superior overall results, surpassing the U-Net model and also for the internal validation dataset. These results suggest the potential of the pix2pix model for placental vessel segmentation and highlight its value for future research and development efforts.

We hypothesized that a cGAN architecture would provide better results for a small dataset than a more general U-Net architecture, as suggested in the literature [13]. Despite the relatively higher computational costs, the significantly higher overall scores indicate a clear benefit of including a discriminative model in the training process, especially regarding generalizability. In addition, GANs have provided impressive results in the field of computer vision and medical imaging regarding efficient data training, image augmentation, and image-to-image translation. Whether fetal surgery can benefit from GANs in the same way and extant as other fields is to be investigated. The relatively low availability of fetoscopic image datasets provides opportunities for GANs or vision transformers, especially [19].

Still, limitations of the experimental protocol may be seen in the model’s architecture, dataset size, and annotated classes. Even though no significant improvements have been seen in recent methods for placental vessel segmentation beyond the standard U-Net and pix2pix, many improved models have been proposed, which may improve the results [20,21,22,23]. Also, inaccuracies were found in the manually annotated labels, which raises the question regarding the absolute ground truth for performance evaluation. For example, in Figure 2, row 3, the presence of specular reflections in the fetoscopic images results in inaccuracies in the segmentation, underscoring the limitation of human-driven annotations. Furthermore, in this study, we focused on the placental vessel, but it might be interesting also to include classes such as inter-twin membrane, fetoscopic tool, or the fetus [11,20,24]. Of course, refinements in architecture may provide benefits regarding the segmentation results. For example, we set the batch size at 1, which may lead to higher computational overhead and slower training due to frequent weight updates. However, the question arises to what extent improvements are still possible for the semantic segmentation task of fetoscopic images and how this influences the development of computer-assisted surgical applications.

## 5. Conclusions

This study focused on the placental vessel segmentation task for fetoscopic images and compared the standard U-Net with a pix2pix model. We were able to reproduce the benchmark results and improve the placental vessel segmentation results by introducing a discriminative model in the training process. In addition, we evaluated the results with an internal dataset, where again, the pix2pix model provided better results. Still, the question of how to achieve generalizability remained unanswered. Nevertheless, these methods can effectively support surgeons in identifying placenta vessels during fetoscopic laser surgery. They may be beneficial in reducing the surgeon’s mental workload, shortening the procedure length, and reducing patient risks.

## Figures and Tables

**Table 1:** Quantitative results of placental vessel segmentation using different models, i.e., the state-of-the-art U-Net and the proposed pix2pix model. Values of the baseline are given in mean and standard deviation, derived from Bano et al. [3]; all other values are given in median and interquartile intervals.

	No. of Validation Images	Evaluation Metrics	Bano et al. (Baseline)	U-Net Model (Reproduced)	Pix2pix Model	Wilcoxon Signed Rank Test (*p*-Value)
Fold 1	120	Dice	0.85 ± 0.07	0.83 [0.74; 0.87]	0.86 [0.82; 0.88]	<0.01
		IoU	0.74 ± 0.10	0.74 [0.64; 0.79]	0.77 [0.73; 0.80]	<0.01
Fold 2	101	Dice	0.77 ± 0.16	0.81 [0.73; 0.85]	0.81 [0.74; 0.86]	0.97
		IoU	0.64 ± 0.17	0.72 [0.64; 0.77]	0.71 [0.64; 0.77]	0.59
Fold 3	39	Dice	0.83 ± 0.08	0.83 [0.78; 0.89]	0.85 [0.78; 0.88]	0.66
		IoU	0.72 ± 0.12	0.74 [0.68; 0.81]	0.75 [0.68; 0.81]	0.47
Fold 4	88	Dice	0.75 ± 0.18	0.51 [0.48; 0.61]	0.72 [0.60; 0.81]	<0.01
		IoU	0.62 ± 0.20	0.49 [0.45; 0.54]	0.63 [0.53; 0.72]	<0.01
Fold 5	37	Dice	0.70 ± 0.18	0.75 [0.62; 0.83]	0.70 [0.67; 0.76]	0.22
		IoU	0.56 ± 0.19	0.65 [0.53; 0.73]	0.62 [0.56; 0.66]	0.05
Fold 6	97	Dice	0.75 ± 0.12	0.72 [0.60; 0.76]	0.73 [0.65; 0.80]	<0.01
		IoU	0.62 ± 0.15	0.62 [0.53; 0.67]	0.63 [0.57; 0.70]	0.01
Overall	483	Dice	0.78 ± 0.13	0.75 [0.60; 0.84]	0.80 [0.70; 0.86]	<0.01
		IoU	0.66 ± 0.15	0.66 [0.53; 0.75]	0.70 [0.61; 0.77]	<0.01
Internal validation dataset	245	Dice	-	0.53 [0.49; 0.64]	0.68 [0.53; 0.79]	<0.01
		IoU	-	0.49 [0.17; 0.56]	0.59 [0.49; 0.69]	<0.01

## Data Availability

The data presented in this study are available on request from the corresponding author. The data are not publicly available due to privacy restrictions.

## Appendix A

### Appendix A.1. Loss Functions

#### Appendix A.1.1. U-Net

The loss function of the U-Net is described as the sum of the binary cross-entropy loss and Jaccard loss during training, which is given by

(A1) $$L\left(p,\hat{p}\right)=L_{bce}\left(p,\hat{p}\right)+L_{jaccard}\left(p,\hat{p}\right),\;=-\frac{\sum [p\left(log\hat{p}\right)=\left(1-p\right)log\left(1-\hat{p}\right)]}{N}+\left[1-\frac{\sum \left(p,\hat{p}\right)+\delta }{\sum \left(p+\hat{p}\right)-\sum \left(p,\hat{p}\right)+\delta }\right]$$

where p and $\hat{p}$ are the flattened ground truth and predicted label tensor, N is the total number of pixels in the image, and δ is an arbitrarily selected variable (set to 10 × 10^−5^) [3].

#### Appendix A.1.2. Pix2pix

The loss function of the pix2pix model is described as a combination of the L1 loss function and conditional adversarial loss, given by

(A2) $$L_{cGAN}\left(G,D\right)=E_{x,y}\left[logD\left(x,y\right)\right]+E_{x,z}[log(1-D\left(x,G\left(x,z\right)\right)]$$

(A3) $$L_{L1}\left(G\right)=E_{x,y}[||y-G\left(x,z\right){\left|\right|}_{1}]$$

(A4) $$L_{pix2pix}=\arg{min}_{G}{max}_{D}L_{cGAN}\left(G,D\right)+L_{L1}(G)$$

where *λ* is a hyperparameter to specify the weight of the L1 error.
